# Capillary drops, capillary pooled, and venous blood samples for determining hemoglobin concentration using HemoCue: A measurement system analysis

**DOI:** 10.1371/journal.pone.0312233

**Published:** 2024-10-25

**Authors:** Ignacio Méndez-Gómez-Humarán, Vanessa De la Cruz-Góngora, Omar Dary, Teresa Shamah-Levy

**Affiliations:** 1 Center for Research in Mathematics CIMAT, Aguascalientes Unit, Aguascalientes, Mexico; 2 Center for Evaluation and Survey Research, National Institute of Public Health, Cuernavaca, Morelos, Mexico; 3 Global Brain Health Institute, Trinity College Dublin, Ireland; 4 USAID, Bureau for Global Health Washington DC, Washington, DC, United States of America; Universitas Padjadjaran, INDONESIA

## Abstract

Several external and internal factors can affect the performance and variability of Hemoglobin concentration [Hb] measurements using HemoCue, and documentation on the contribution of different sources of [Hb] variation is limited. We used an experimental repeated measurements design with nine randomly selected participants, three HemoCue devices, and three trained field workers. HemoCue measurements for all samples were repeated three times. The [Hb] measurement system considers four sources of error: 1) HemoCue devices, 2) field workers, 3) between individuals, and 4) within individuals. A concordance analysis was used to assess accuracy and precision, and a linear mixed model was used to estimate mean differences (bias) from blood specimens, anticoagulants, and to estimate the contribution of the 4 sources of error to [Hb] measurements. Positive mean [Hb] differences were found: 1.34 g/dL for capillary drops, 0.81 g/dL for pooled capillary blood samples, 0.756 g/dL for venous blood stored with EDTA, and 0.911 g/dL for venous blood stored with heparin. The mean [Hb] difference for venous blood with EDTA was used as a correction factor for all results measured using a HemoCue. After adjustment, capillary drops showed a mean difference of 0.585 g/dL, and pooled capillary samples were not significantly different. The individual variability was 95.8% of total variance, HemoCue devices contributed 2.1% of measurement error, field staff contributed 0.4%, and the residual was 1.7%. The HemoCue [Hb] measurement system is reliable in controlled environments, with a small measurement error of 4.2%.

## Introduction

Recent evidence has shown inconsistencies in anemia prevalence across different surveys, which highlights important variations in hemoglobin concentration [Hb] measurements [1, 2]. Rappaport et al. documented [Hb] variation, but could not distinguish whether these differences were attributable to the type of blood, the HemoCue model used to measure, or the sampling techniques [3]. Larson et al. [4] documented high variability of [Hb] in capillary blood samples versus venous blood. Using HemoCue devices (201+ and 301+), Hruschka et al. reported a notable difference in the prevalence of anemia reported when measured using drop capillary *versus* venous blood [1]. As differences have been documented in the field around sample handling and data management techniques when measuring Hb (e.g., temperature, technique of blood sampling extraction, type of anticoagulant, equipment used, etc.) [5, 6], it is important to assess the source of measurement errors to adjust or to control them in the standardization stage.

Validation studies of hemoglobin concentration using point-of-care devices (including HemoCue) should be an important component of any nutritional survey conducted in a low resource environment. Nevertheless, validation of Hb data, in most studies, if performed, mostly relies on bias adjustment [7, 8], and the analysis of the [Hb] variation and its components are poorly documented [3, 9, 10]. There are a broad range of known sources of variation: for example, different blood sampling techniques such as capillary drop, pooled drop or venous blood, age groups, staff training, and external factors such as temperature and humidity.

The measurement process may have several sources of variation. These include natural variation amongst the subjects being measured, as well as variation in field procedures for blood sampling or HemoCue devices used, any of which may contribute to measurement error and produce incorrect inferential results.

Measurement systems analysis (MSA) is a method that identifies the components of variation in the measurement process. An MSA evaluates the measuring instruments, the sampling procedure, and the entire process of obtaining measurements to ensure the integrity of the data used for analysis and understand the implications of measurement error on the ability to make inferences from a sampling procedure.

In this context, analysis of the reliability of [Hb] measurements based on capillary samples using portable photometers as HemoCue, and the contribution of different variability sources in hemoglobin measurement errors has become an important area of research.

Therefore, this study aimed to analyze the accuracy and precision of hemoglobin measurements, including: 1) assessing the bias contribution of two capillary blood sampling techniques (drop and pool) and two different anticoagulants (ethylenediaminetetraacetic acid [EDTA], and heparin) for storage of venous blood samples, and 2) evaluating the contribution of the Hemocue 201+ device and sampling staff to [Hb] measurement error using an MSA approach.

## Methods

In industry and for proper control of processes and products, measurement systems have become a critical issue and require a distinct methodological approach [11–13]. Experimental designs focus on the variability of elements of the measurement process, built with a specific intention to evaluate measurement reliability and not to produce inferential results about statistical samples. Sample sizes are planned to measure all sources of variability; therefore, they often use small samples and repeated measures at each step of the process. Here, a repeated-measurement experimental design was used to separate true individual variation from other error sources, including across HemoCue devices, between blood sampling procedures, and between staff members, using Gage Repeatability and Reproducibility (Gage R&R) approach. Gage R&R is a methodology used to define the amount of error in the variation of measurement data [14]. The design considered two components of reliability: 1) the bias contributed by two blood sampling methods and two anticoagulant storage media for the venous samples, and 2) the measurement error contributed by identifiable sources of variability for the [Hb] measurement system through a Gage R&R type design. Nine adults aged 18–49 years were included, both male and female, with a hemoglobin concentration over 8 g/dL. Exclusion criteria included patient-reported active fever or cold symptoms, suspected COVID or COVID exposure, current pregnancy or lactation, a history of cancer or chemotherapy, a previous mastectomy, or a history any disease related to hematological disorders.

**Fig 1:**
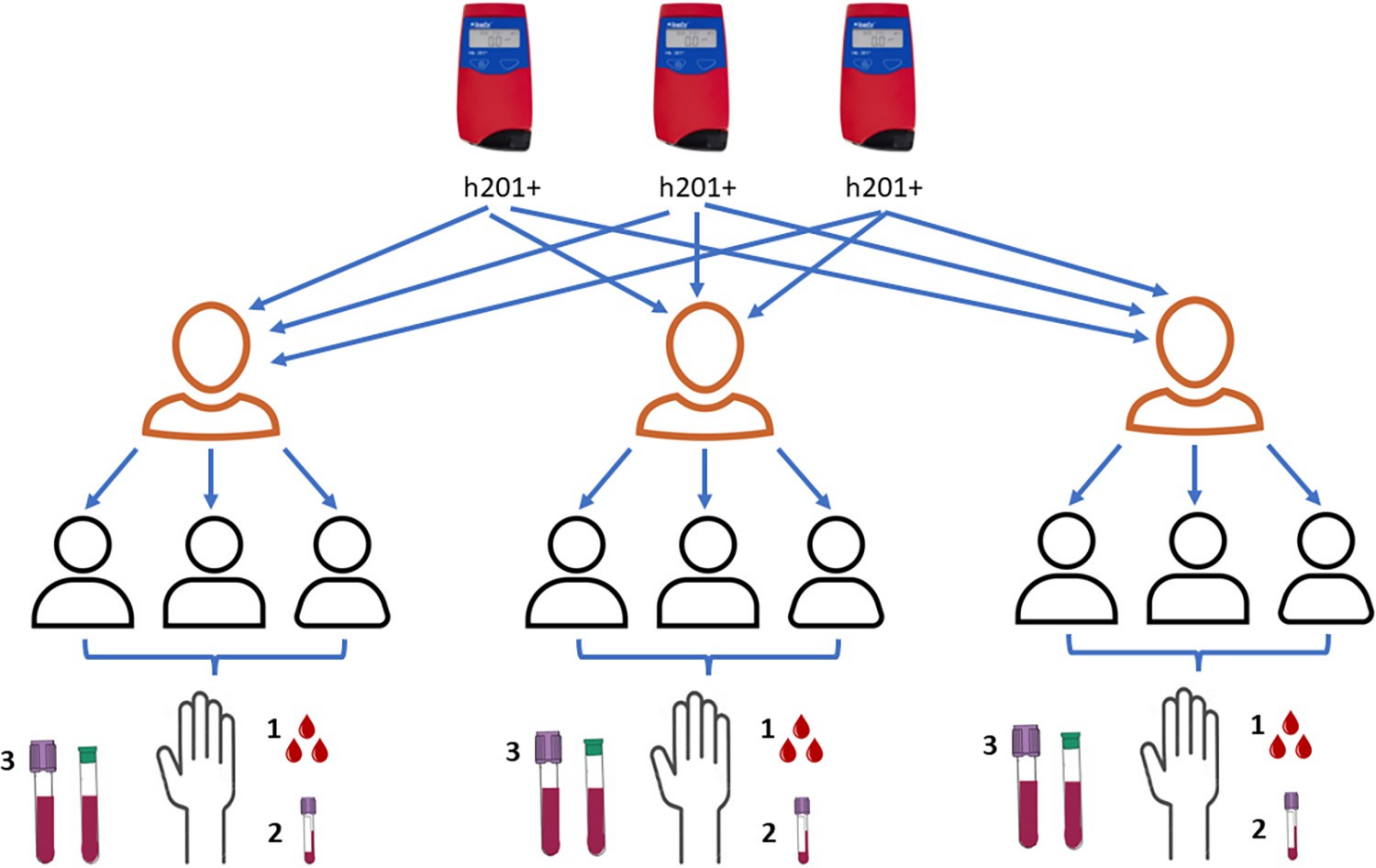
Experimental design for hemoglobin measurements. *Numbers are the order of procedures done to each participant*.

Three trained staff were responsible for collecting blood samples from three different participants. Each staff member used a HemoCue 201+ device (HemoCue. Hb 201, Angelholm, Sweden) (n = 3) for each corresponding subject (n = 3/9); therefore, the HemoCue device and staff were both included as crossed factors. The design considered four sources of identifiable variability for the [Hb] measurement system. The first source of variation was between HemoCue devices, the second was between staff members, the third was between subjects (natural variability), and the fourth was the within-subject residual error from repeated measurements on the same individual. Fig 1 shows the experimental design diagram for the [Hb] measurements.

### Blood collection procedures and laboratory measurements

Three direct drops of blood (drop sample) were collected from subjects through a fingertip on the left hand using high-flux BD lancets (blue color: Mexican catalog key: 080.574.0032, code number: 366594), where the first drop of blood was wiped away with a sterile 2 × 2 gauze. The three subsequent drops were placed in three microcuvettes (batch: 1903391) to measure [Hb] using a HemoCue. The fifth and subsequent drops were placed in microtainer tubes with K_2_-EDTA (code number: 365974) to make 350–500 mcL. After mixing, a sample from the capillary blood pool was collected and placed in three microcuvettes to measure [Hb] with a HemoCue (pool sample). Then, 3 mL of venous blood was collected from the left arm in Vacutainer tubes with one of two anticoagulants: EDTA (BD, code number 368171), or heparin. After mixing, a sample from the venous blood pool from each Vacutainer tube (EDTA and Heparin) was taken, and three drops were placed in three microcuvettes to measure [Hb] with a HemoCue. The remaining capillary and venous blood collected in microtainer and Vacutainer tubes with EDTA were sent to the central laboratory (Centro Hematológico de Morelos, Mexico) within the subsequent 4 hours for [Hb] analysis with the Beckman Coulter Ac•T 5diff® (Beckman Coulter Inc., Danaher Corporation, California, USA) using the cyanmethemoglobin method [15]. This measurement was considered the gold standard.

### Statistical analysis

We assessed two central components of analysis. The first is related to the equivalence of measurements. Concordance analysis was used to evaluate the agreement between hemoglobin measurements from blood samples based on both capillary methods and venous samples stored with EDTA or Heparin obtained through the HemoCue, in contrast to venous blood measurements using the gold standard. The concordance correlation coefficient measures precision and accuracy simultaneously, using the Pearson correlation coefficient as a measure of precision and relative bias as a measurement of accuracy. The Bland–Altman (BA) mean difference is an estimate of the absolute bias between the HemoCue measurements and the gold standard. The mean difference in EDTA-stored venous samples was used as a correction factor for all capillary blood samples, based on the idea that the mean difference represents a measurement bias associated with the use of EDTA as a storage medium for blood samples, and a concordance correlation analysis was applied to the adjusted measurements.

A variance ratio test was used to compare variances from the BA differences, as precision of [Hb] measurements, between the drop and pool-based capillary sampling techniques.

The second component of analysis evaluated the contribution of the variability sources of interest to [Hb] measurement errors; thus, an MSA was generated using a linear mixed model. The fixed effects of the model include a four-level factor considering the two capillary blood sampling procedures and two venous blood sampling storage methods, which enables bias estimation of [Hb] and comparison of the capillary blood sampling methods. The random effects included the four variation sources of the hemoglobin measurement system (variance components) and were used to identify the contribution sizes of variability sources to the measurement system errors.

Concordance correlation coefficients and BA mean differences were calculated using *Stata Statistical Software* (Release 16. College Station, TX: StataCorp LLC., USA). The linear mixed model analysis was generated using SAS/MIXED software on SAS Studio Version 3.8 Copyright © 2012–2018 SAS Institute Inc. on the SAS OnDemand for Academics online platform.

### Ethics

The study protocol was approved by the Research Ethics and Biosecurity Committees of the National Institute of Public Health of Mexico. A signed informed consent letter was obtained from all participants. Approval number: 1652

## Results

Positive BA mean differences were found between capillary and venous blood specimens measured with the HemoCue 201+ in comparison with venous blood assessed with the gold standard (Table 1). A positive mean difference of 1.34 g/dL was observed for capillary drops: higher than the 0.81 g/dL mean difference from pooled capillary samples.

**Table 1:** Concordance analysis and Bland-Altman mean [Hb] difference.

	Sample HemoCue	n	Concordance	Pearson Correlation	Relative Bias	Mean difference	SD
**Before adjustment**							
Drop Capillary	Drop	27	0.687	0.958	0.717	1.341	0.462
	Drop 2	9	0.696	0.973	0.715	1.3	0.357
	Drop 3	9	0.693	0.969	0.715	1.356	0.433
	Drop 4	9	0.672	0.938	0.716	1.367	0.612
Pooled Capillary	Pool	27	0.833	0.962	0.866	0.811	0.411
	Pool (drop 1)	9	0.846	0.976	0.867	0.811	0.341
	Pool (drop 2)	9	0.841	0.96	0.876	0.789	0.451
	Pool (drop 3)	9	0.81	0.951	9.852	0.833	0.477
Venous pool	Venous heparin	27	0.829	0.983	0.843	0.911	0.294
	Venous EDTA	27	0.874	0.896	0.887	0.756*	0.282
*correction factor							
**After adjustment**							
Drop Capillary	Drop	27	0.889	0.958	0.928	0.585	0.462
	Drop 2	9	0.91	0.973	0.935	0.544	0.357
	Drop 3	9	0.896	0.969	0.925	0.6	0.433
	Drop 4	9	0.865	0.938	0.92	0.611	0.612
Pooled Capillary	Pool	27	0.961	0.962	0.99	0.055	0.411
	Pool (drop 1)	9	0.975	0.976	0.99	0.055	0.341
	Pool (drop 2)	9	0.959	0.96	0.99	0.033	0.451
	Pool (drop 3)	9	0.948	0.951	0.99	0.07	0.477
Venous pool	Venous heparin	27	0.976	0.983	0.99	0.155	0.294
	Venous EDTA	27	0.983	0.986	0.99	0	0.282

The difference using venous blood with EDTA was 0.756 g/dL *versus* with heparin was 0.911 g/dL. The [Hb] mean difference observed in venous blood with EDTA was used as the HemoCue bias correction factor for all results obtained with HemoCues. After adjustment, capillary samples showed a positive mean difference of 0.58 g/dL with a concordance correlation coefficient of 0.889, considering a 0.928 relative bias. In comparison, pooled capillary samples had a non-significant difference (0.05 g/dL) with a concordance of 0.961, and demonstrated a negligible relative bias of 0.99 (1.0 is perfect identity or non-bias). For heparin-treated venous samples, the mean difference was 0.155, with negligible bias.

For the sequential order of individual drops, the concordance correlation, Pearson correlation, and the relative bias were quite similar amongst all three drops. This is evidence that the selection of the second to the fourth does not influence the measurement variability.

**Fig 2:**
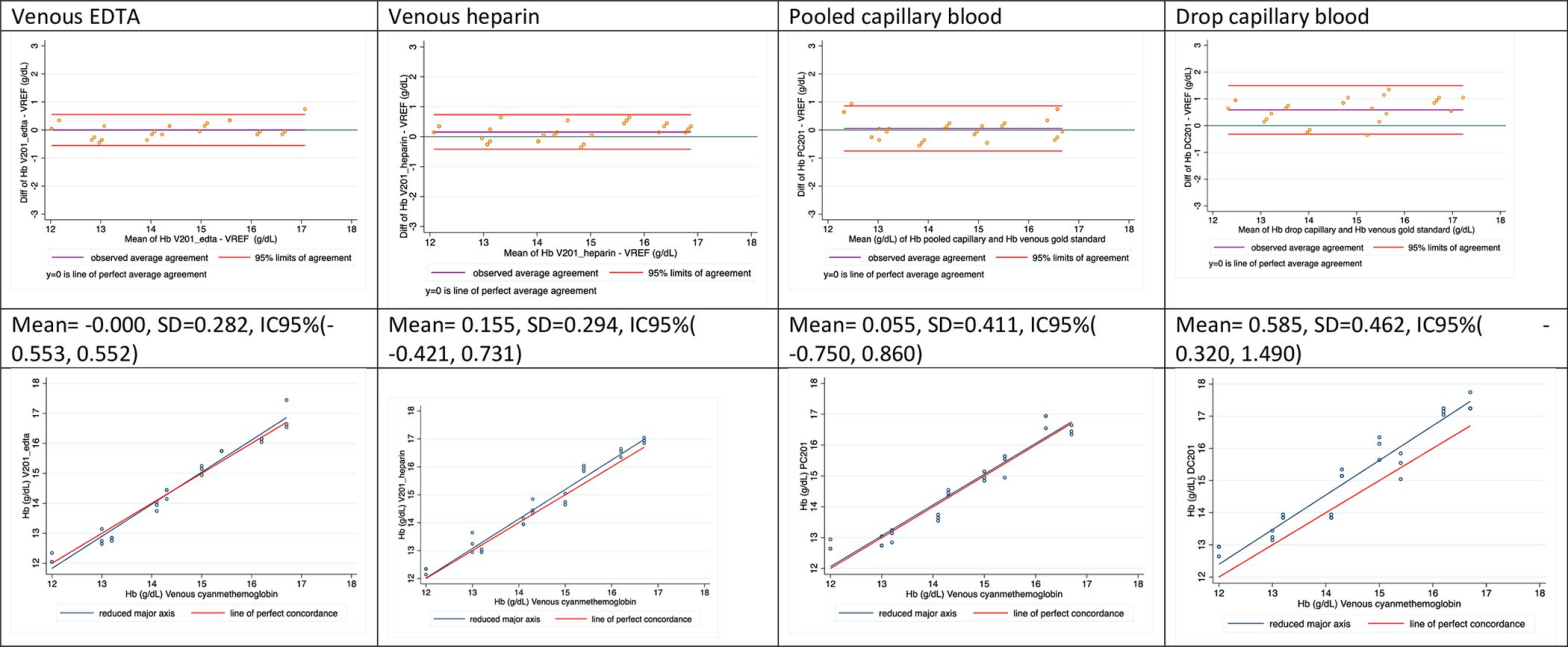
Bland Altman and concordance plots of blood samples analyzed in HemoCue 201+* vs Cyanmethemoglobin method. VREF: [Hb] measured in venous blood using cyanmethemoglobin in an Automated Hematology Analyzer; V201_EDTA: [Hb] measured in venous blood mixed with EDTA using HemoCue 201+; V201_heparin: [Hb] measured in venous blood mixed with heparin using HemoCue 201+; PC201: [Hb] measured in pooled capillary blood using HemoCue 201+; DC201: [Hb] measured in drops of capillary blood using HemoCue 201+.

The first row of Fig 2 shows the BA estimated mean difference (agreement) and 95% confidence interval, where a significant positive bias is evident for capillary drop samples. Dispersion was higher in both capillary samples than venous blood using HemoCue; a variance ratio test indicated that drop-based [Hb] measurements were not statistically different from pool-based [Hb] measurements (*p* = 0.979). In fact, the standard deviations for BA limits were very similar: 0.346 for drop samples and 0.345 for pool samples.

The second row of Fig 2 shows the concordance correlation represented as a linear correspondence between the [Hb] measurements and the gold standard, where only capillary drop samples are slightly biased.

Tables 2 and 3 show the results of the linear mixed model for the corrected measurements. Table 2 shows fixed effect estimates, where the intercept represents the mean [Hb] measured using the gold standard (14.4 g/dL). The coefficients represent mean differences (bias) estimates for all samples, where drop samples showed a positive bias of 0.58 g/dL (p<0.022) and pool samples showed a negligible bias of 0.056 g/dL (p = 0.803). Drop capillary samples showed a significantly higher bias than pooled capillary samples (p = 0.015, not shown in the table). Venous samples stored in heparin showed a non-significant bias of 0.156 g/dL (*p* = 0.49) as compared to the gold standard.

**Table 2:** Fixed effects estimate for sampling methods on [Hb] measurement after bias adjustment*.

Fixed effect estimates	Coefficient	Std. Error	Prob>|t|
Intercept	14.433	0.550	< .0001
Drop samples	0.585	0.217	0.022
Pool samples	0.056	0.217	0.803
EDTA venous samples	0.000	0.217	1.000
Heparin venous samples	0.156	0.217	0.490

**Table 3:** Variance components of the measurement system.

Random effect estimates	Variance components	Standard error	Pct of total variance
Inter-HemoCue	0.0555	0.0277	2.1%
Sampling personnel	0.0101	0.0103	0.4%
Subject	2.5104	1.2727	95.8%
Residual	0.0440	0.0075	1.7%
Total	2.6200	1.2690	100%

Table 3 shows the variance component estimates for the four measurement sources of variation. The subject (person providing the blood sample) was considered a source of natural variation and was independent of the measurement system, with 95.8% of the total system variance. The Hemocue 201+ device variance represented 2.1% of the total variation, laboratory staff accounted for 0.4% of the variation, and the residual variance was 1.7% of the total system variance. These results reveal that the system variance represents 4.2% of the total system variance (2.1+1.7+0.4). Therefore, when using well-trained staff and pooled blood sample techniques for blood specimens, the HemoCue-based measurement system produces reliable results in a controlled environment.

## Discussion

In this controlled trial, capillary pool samples showed lesser bias and better performance in Hb measurement as compared to single-drop samples, although both had similar variances and precision. Nevertheless, some authors [16, 17] have discouraged the use of capillary drops because of the notable variation which affects interpretation of hemoglobin values. Previously, we documented and discouraged the use of capillary drops because of high variation in [Hb] data, particularly in children, as compared to [Hb] variation from pooled capillary and venous blood [15]. The use of capillary blood drops can be more sensitive to external factors in field studies, which may increase the variance of measurements. This leads to the selection of pooled samples as the recommended capillary sampling technique for field studies.

Measurements using pooled capillary blood samples with a HemoCue-based system can be reliable when bias corrections are properly investigated and implemented. The use of capillary samples is practical and inexpensive in the field, but requires that the contributing factors to measurement errors be controlled.

In terms of the error components within the measurement system, we found that the differences inherent to the sampled individuals represented the majority of the observed variability in the measurements. The HemoCue 201+ device and sampling staff contributed minimally to the system measurement error, representing together less than 5% of the overall variability.

In field work, well-trained and standardized staff are crucial factors that minimize both potential errors in the determination of [Hb] and variation attributable to the HemoCue device [16]. An error component of less than 30% of total variance is considered a good measurement system [13, 14]. In traditional sampling rationale, sample size is relevant to ensure low error in prevalence estimation, while small sample sizes are useful to separate the individual variation from other error sources.

As many additional sources of Hemoglobin variation exist—for example, individual aspects such as age and gender, environmental factors, sampling techniques, the HemoCue device, the staff taking blood samples, the anticoagulant used, and others—these sources should also be considered and documented. In our study, comparison of EDTA and heparin was not significant, both showed a similar bias which may have been related to the HemoCue device. EDTA as an anticoagulant was used as a standard reference, since it is widely used, and was also considered the gold standard. The difference used as the correction factor was the HemoCue bias estimation, and the heparin bias was not significant from the corrected measurements; therefore, heparin and EDTA can be used with similar confidence level.

This study had several limitations that should be considered when interpreting the results. Controlled trials are centered on the analysis of the measurement system and cannot be extrapolated to field studies because of individual heterogeneity, living conditions, and environmental factors such as temperature and humidity, which can increase measurement error [5].

In addition, the selected subjects were not anemic; subjects with lower values of [Hb] were not considered for ethical reasons. It is possible that the estimated bias related to capillary samples using HemoCue change due to a wider [Hb] heterogeneity in sampled individuals.

Another limitation is that the bias and the error contribution of the gold standard method are assumed to be zero. Ideally, experimental design models should consider other certified laboratories to check for a possible bias, and conduct repeated measurements using the gold standard to estimate the marginal error of the cyanmethemoglobin [Hb] measurements. These errors can contribute to the bias estimate and random variability of [Hb] measurement.

Validation of the HemoCue should be routinely performed before data collection in field settings. We recommend the use of experimental designs with an MSA to calibrate the Hb measurements in fieldwork and evaluate the contribution of common sources of variation and other potential factors, such as weather and aspects of blood sampling, to validate the reliability of population survey samples. This allows standardization and procedural improvements to measurement of [Hb] in epidemiological studies. For example, if the error related to technicians becomes higher, we can detect it and establish corrective procedures. Additionally, there exists the possibility of emerging bias and an increase in measurement error due to equipment wear on the HemoCue devices. Common procedures used to validate and calibrate [Hb] measurements (e.g., Bland and Altman, linear regression, etc.) are useful for bias correction and measure the size of the total measurement error, but ignore elements of blood sampling and [Hb] procedures that contribute to the total measurement error. However, MSA can separate and evaluate the error contribution of each part of the sampling and measurement procedures.

## Conclusions

In a controlled setting, the [Hb] measurement system was reliable; Hb from capillary samples (drop and pool) showed similar performance using the HemoCue, but pooled samples had a lower bias than drop samples. The bias of [Hb] measurements using HemoCue for venous blood samples stored with EDTA or heparin was similar. Use of the HemoCue 201+ is reliable for bias-corrected measurements in a controlled setting, but requires sound training procedures for staff to maintain a robust Hb measurement system in field studies.
